# Gallbladder Volvulus: A Necessary Consideration When Managing Cholecystitis Conservatively

**DOI:** 10.7759/cureus.87080

**Published:** 2025-06-30

**Authors:** Anna Mealy, Mikhail Fisher

**Affiliations:** 1 General Surgery, Monash Health, Melbourne, AUS

**Keywords:** cholecystitis, gallbladder volvulus, geriatric surgery, hepatobiliary, laparascopic surgery, laparosocpic cholecystectomy, non-specific abdominal pain

## Abstract

Gallbladder volvulus is a rare surgical pathology predominantly affecting an older patient profile, in which frailty and comorbidities play an important role in surgical decision-making. The difficulty in accurately diagnosing this condition lies in the vague, non-specific symptoms and the lack of a reliable radiological diagnosis. This case report describes the management of gallbladder volvulus in a 91-year-old female and highlights the fact that antibiotics alone will not manage this pathology. Therefore, this condition must be part of the differential diagnosis when considering conservative management of cholecystitis.

## Introduction

Gallbladder volvulus is a rare disease occurring in 1 out of 365,520 cases of gallbladder disease, predominantly affecting elderly women [1]. The exact cause of gallbladder volvulus is unknown, but a variant in anatomy allowing the gallbladder to twist around a pedicle of mesentery is thought to predispose to torsion [2]. In adults, it is thought that these congenital variants are exacerbated by physiological changes associated with aging, such as weight loss and liver atrophy [3]. Due to the potential involvement of the cystic artery in the volvulus, there is a high risk of gallbladder ischemia and necrosis, and timely surgical intervention is required to ensure recovery. This may prove challenging as preoperative diagnosis is rare, with 75% of the diagnoses made intraoperatively [4]. The lack of an accurate diagnosis may result in delays to the theater, as acute cholecystitis (gallbladder inflammation, most commonly caused by gallstones) may be treated less urgently.

A systematic review reported that 76% of cases were in the adult population, with a median age of 77, and 79% of adult patients were female [5]. In this cohort of patients, where frailty and comorbidities play a role in the decision of surgical management versus conservative management [6], gallbladder volvulus needs to be appreciated as a potential differential diagnosis, as this pathology will not improve with antibiotics or the insertion of a cholecystostomy tube [7,8].

## Case presentation

A 91-year-old female presented to the emergency department with a one-day history of right upper quadrant pain. This was the first episode of such pain, with no other associated symptoms. She had a significant medical history of atrial fibrillation, for which she was on apixaban. Her last dose was taken the morning of presentation. Other relevant history included a single-chamber pacemaker, hypertension, and dyslipidemia.

Upon presentation, she was hemodynamically stable with a heart rate of 74 beats/minute, a blood pressure of 165/91 mmHg, and a temperature of 36°C. Her abdomen was soft but tender in the epigastrium and right upper quadrant, with a positive Murphy’s sign. Table 1 shows a summary of the blood results on admission and the day of operation. White blood cells (WBCs) were found to be raised, but C-reactive protein (CRP) and liver function tests were unremarkable (Table 1).

**Table 1:** Laboratory results on presentation and the day of theater admisstion. A rise in white blood cells and C-reactive protein can be seen from the day of admission to the day of theater admission, despite the patient being on appropriate antibiotics.

Blood analysis	On admission	Day of theater	Reference range
White blood cells	14	20.2	4.5–11 × 10^9^/L
C-reactive protein	1	245	<5 mg/L
Alanine aminotransferase	11	-	3–40 IU/L
Alkaline phosphatase	60	-	30–100 IU/L
Gamma-glutamyl transpeptidase	24	-	8–60 IU/L
Bilirubin	14	-	3–17 µmol/L

**Figure 1:**
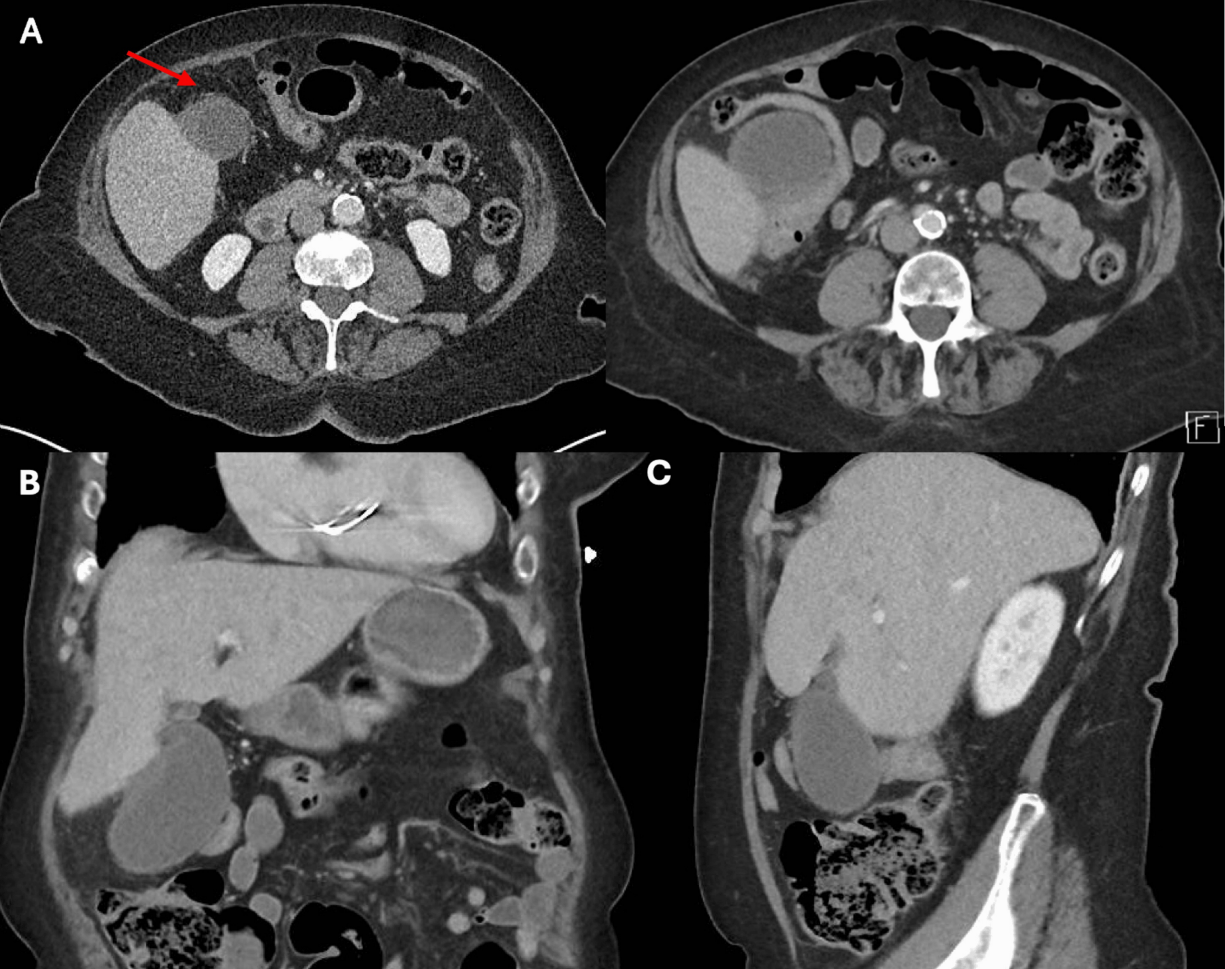
Contrast-enhanced CT of the abdomen and pelvis showing a distended gallbladder. A thickened gallbladder wall measuring 4 mm was noted. Mild fat stranding can be seen along the body of the gallbladder. No radiopaque cholelithiasis was noted. A. Axial images of the gallbladder showing mild fat stranding (arrow). B. Coronal image of the gallbladder showing a distended gallbladder and gallbladder wall thickening. C. Sagittal image of the gallbladder showing a distended gallbladder.

Contrast-enhanced CT of the abdomen and pelvis was performed on presentation to the emergency department before a referral to the general surgical team. It showed a distended gallbladder with wall thickening and surrounding fat stranding suspicious for cholecystitis (Figure 1).

**Figure 2:**
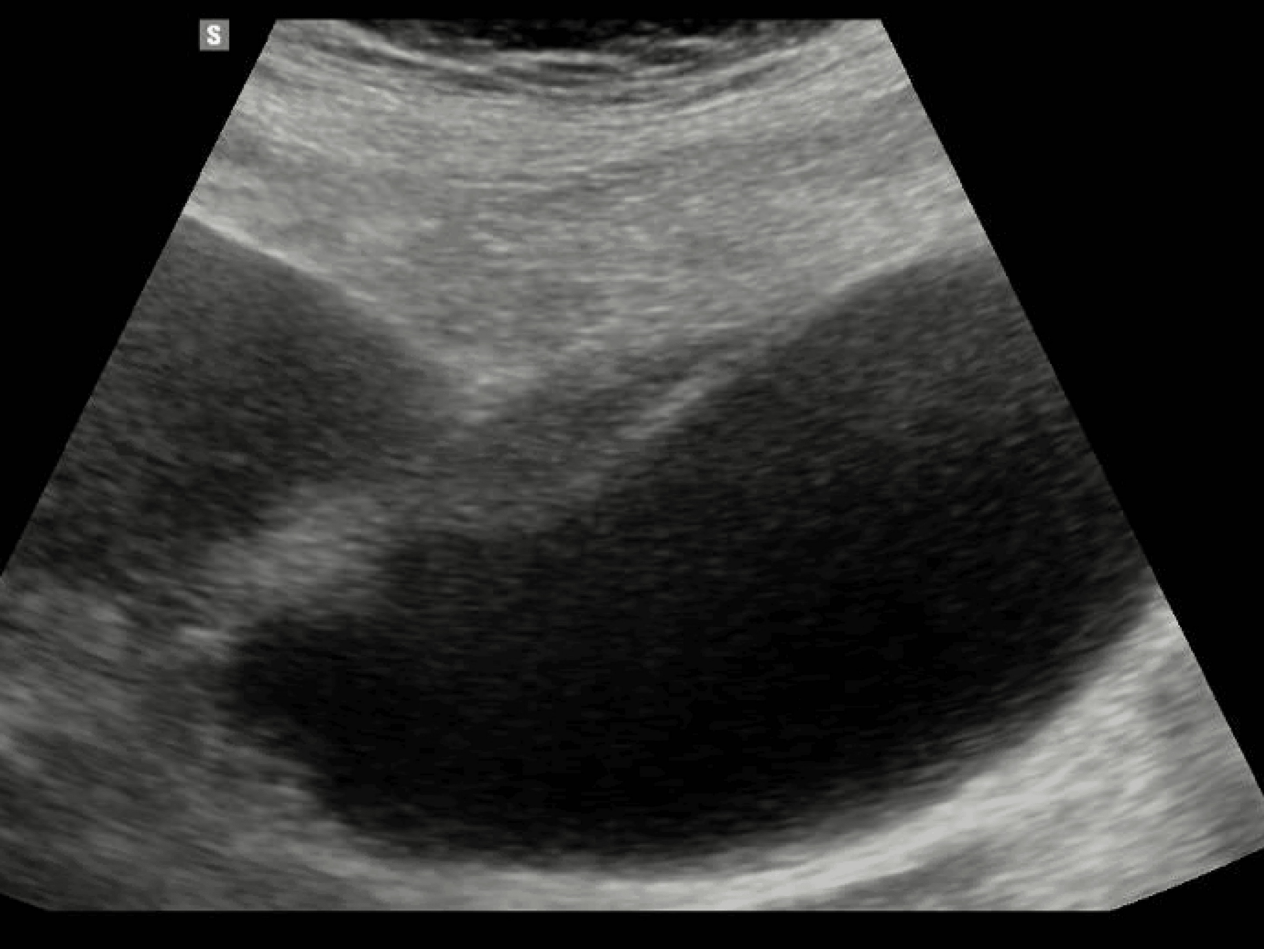
Ultrasound of the gallbladder showing a distended gallbladder. Symmetrical wall thickening of 8 mm with mild vascularity and evidence of pericholecystic fluid was noted. There was marked focal tenderness over the gallbladder. No gallbladder calculi were seen.

An ultrasound performed on the same day showed a distended gallbladder with wall thickening of 8 mm and mild vascularity (Figure 2). The was evidence of pericholecystic fluid and marked focal pain over the gallbladder. No gallbladder calculi were visualized, nor was there any biliary duct dilation; the common bile duct measured 3.2 mm.

A radiographical diagnosis of acalculous cholecystitis was made, and the patient was started on intravenous (IV) ceftriaxone as per hospital guidelines. As an underlying cause for acalculous cholecystitis could not be found, such as heart failure or another cause of sepsis, a decision was made for operative management. Apixaban was withheld, and a medical review was requested for preoperative optimization.

The theater was planned for 48 hours after admission to allow for appropriate washout of apixaban. The CRP reached a maximum of 245 mg/L, and WBC reached a maximum of 20.2 × 10^9^/L. While the patient remained afebrile, she became tachycardic with a heart rate of 98 beats/minute and a decreasing blood pressure of 105/73 mmHg on the day of theater admission. She developed an ileus with increasing distension. The patient progressed to the theater as planned, and a laparoscopic cholecystectomy was performed.

**Figure 3:**
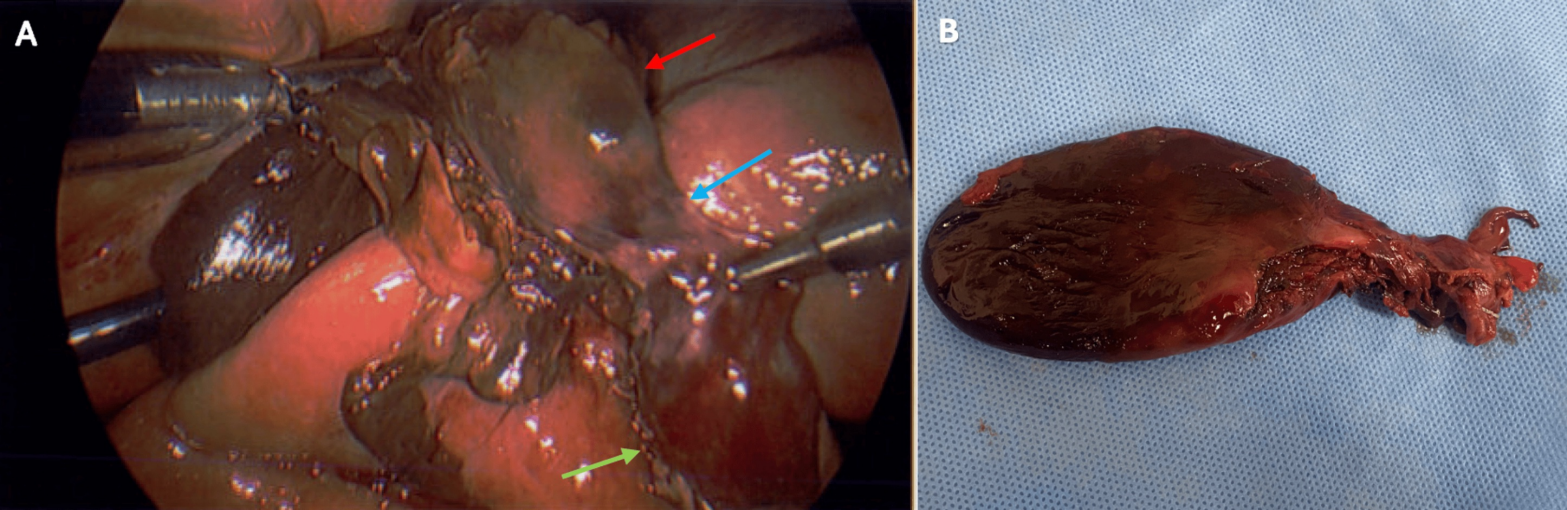
Intraoperative findings of the laparoscopic cholecystectomy and postoperative specimen. A. Intraoperative findings of a gallbladder volvulus. The red arrow shows the distended, gangrenous body and fundus of the gallbladder. The green arrow shows viable neck of the gallbladder. The blue arrow highlights area of torsion beneath inflammatory adhesions. B. Postoperative specimen, showing the gangrenous body and fundus of the gallbladder.

A diagnosis of gallbladder volvulus was made intraoperatively. A distended, gangrenous body and fundus of the gallbladder were visualized with torsion, leaving a viable neck (Figure 3). A moderate amount of bile spillage occurred intraoperatively due to the inadvertent perforation of the gallbladder during retraction. The operation was completed without complications. The intraoperative cholangiogram was performed as per the hospital’s standard practice, which showed good flow of contrast to the hepatic ducts and the duodenum. A drain was placed in the gallbladder fossa.

IV antibiotics were continued for 24 hours postoperatively. The drain tube was removed 48 hours postoperatively, and apixaban was recommenced 72 hours postoperatively, on the day of discharge. The patient was seen in the outpatient clinic three weeks later. She had some mild ongoing right upper quadrant pain, but had generally returned to baseline. The histopathology result confirmed acute-on-chronic cholecystitis with areas of gangrenous cholecystitis.

## Discussion

This case report highlights the challenges that arise with a pathology such as gallbladder volvulus. The symptoms are non-specific, mainly including right upper quadrant pain, nausea, vomiting, and a fever [9], and are usually difficult to distinguish from acute cholecystitis or acalculous cholecystitis. In addition, inaccurate radiological diagnosis may delay the appropriate management by misleading surgeons regarding the correct diagnosis [10].

In this case, a radiological diagnosis of acalculous cholecystitis was made. Acalculous cholecystitis is typically seen in patients who are critically ill, following cardiac surgery, abdominal vascular surgery, severe trauma, burns, prolonged fasting, total parental nutrition, or sepsis [11]. This pathology is more often treated conservatively with antibiotics or, in patients who are less stable, a cholecystostomy tube or biliary stenting via endoscopic retrograde cholangiopancreatography may be considered [12].

While a diagnosis of acalculous cholecystitis was made, many of the features of acalculous cholecystitis were not present. The incongruity between the patient’s presentation and the radiologic diagnosis led to a decision to proceed to surgery early during the patient’s admission. This highlights the importance of carefully correlating patient presentation and history with radiographic investigations. It also highlights the complex nature of managing a disease that is often not diagnosed preoperatively or that is entirely misdiagnosed.

Despite the commencement of the appropriate antibiotics in a prompt manner, a clear deterioration of the patient can be seen during the initial course of the hospital admission before the operation. The WBC and CRP increased to concerningly high levels, and the patient became tachycardic and hypotensive on the day of the operation. Antibiotics alone were clearly failing for this patient.

Prolonging conservative management of a gallbladder volvulus increases the risk of worsening sepsis. In this case, the distal end of the gallbladder was necrotic, and with further delay, tissue necrosis may have progressed, leading to frank gallbladder perforation, worsening biliary peritonitis, and progressive systemic sepsis, all of which would have increased the risk of postoperative morbidity and mortality [13].

## Conclusions

As surgeons, it is our responsibility to carefully consider the age, comorbidities, and frailty of a patient before an operation. It is also our due diligence to ensure that a correct diagnosis is obtained when deciding on conservative management of cholecystitis. As gallbladder volvulus is an intraoperative diagnosis, careful consideration needs to be given to radiological diagnoses that do not match the history or presentation of a patient. This case highlights the difficulty in managing elderly patients presenting with complex biliary conditions and the need for vigilance in considering rare entities such as gallbladder volvulus, which will not resolve with conservative management.
